# A Thorny Tale of Parasites: Screening for Enteric Protozoan Parasites in Hedgehogs from Portugal

**DOI:** 10.3390/ani14020326

**Published:** 2024-01-21

**Authors:** Sara Gomes-Gonçalves, Sérgio Santos-Silva, Andreia V. S. Cruz, Clarisse Rodrigues, Vanessa Soeiro, Patrícia Barradas, João R. Mesquita

**Affiliations:** 1 Department of Biology, Campus de Santiago, University of Aveiro, 3810-193 Aveiro, Portugal; saragomes2000@hotmail.com; 2 School of Medicine and Biomedical Sciences (ICBAS), University of Porto, 4050-313 Porto, Portugal; up202110051@edu.icbas.up.pt (S.S.-S.); up201303679@edu.icbas.up.pt (A.V.S.C.); 3 Centro de Recuperação e Interpretação do Ouriço—CRIDO, 4470-372 Maia, Portugal; crido.amigospicudos@gmail.com; 4 Parque Biológico de Gaia, 4430-812 Vila Nova de Gaia, Portugal; vanessasoeiro@cm-gaia.pt; 5 1H-TOXRUN—One Health Toxicology Research Unit, University Institute of Health Sciences, Cooperativa de Ensino Superior Politécnico e Universitário, CRL(CESPU, CRL), 4585-116 Gandra, Portugal; patricia.barradas@iucs.cespu.pt; 6 Epidemiology Research Unit (EPIUnit), Instituto de Saúde Pública da Universidade do Porto, 4050-600 Porto, Portugal; 7 Laboratory for Integrative and Translational Research in Population Health (ITR), 4050-600 Porto, Portugal

**Keywords:** *Cryptosporidium* spp., *Giardia duodenalis*, *Blastocystis* sp., *Balantioides coli*, hedgehog, Portugal

## Abstract

**Simple Summary:**

This study represents the first investigation of enteric protozoan parasites in hedgehogs in mainland Portugal, focusing on prevalent parasites, including *Blastocystis* sp., *Balantioides coli*, *Cryptosporidium* spp., and *Giardia duodenalis*. *Blastocystis* and *Cryptosporidium* were detected with an occurrence of 0.91% (1/110, 95% CI: 0.02–4.96). These findings highlight the need for continued research and surveillance and mark the first report of *Blastocystis* and *Cryptosporidium* occurrences in hedgehogs of Portugal, providing valuable data to address the occurrence and distribution of these parasites. The detection of parasites with zoonotic potential raises concerns about potential transmission to humans, emphasizing the necessity for continuous monitoring. This study suggests the importance of additional research with larger sample sizes and a wider geographical representation. This can contribute to the development of prevention and control strategies, aiming to better understand and manage potential risks in hedgehog populations and their interaction with other animals and humans.

**Abstract:**

Enteric protozoan parasites, such as *Blastocystis* sp., *Balantioides coli*, *Cryptosporidium* spp., and *Giardia duodenalis*, may have implications for both animal and human health.Transmitted through the fecal–oral route, these parasites cause symptoms such as diarrhea, abdominal pain, and weight loss. This study investigated the presence of these enteric protozoan parasites and genetically characterized them in hedgehogs from Portugal. A total of 110 hedgehog stool samples were collected. Molecular detection methods showed an overall occurrence of protozoa in 1.82% (2/110 95% CI: 0.22–6.41) of hedgehogs, with *Blastocystis* being found in one hedgehog and *Cryptosporidium* being found in another. No evidence for the presence of *B. coli* or *G. duodenalis* was found. This study suggests that there is a need to stay aware of hedgehogs as potential hosts of enteric protozoa. Ongoing research and surveillance efforts are recommended to explore practical prevention and control strategies. The results contribute to the limited knowledge of these parasites in Portuguese hedgehog populations and underscore their potential relevance to both veterinary and public health.

## 1. Introduction

*Blastocystis* sp., *Balantioides coli*, *Cryptosporidium* spp., and *Giardia duodenalis* are common enteric protozoan parasites that share the same fecal–oral infection route and can pose a threat to the health and well-being of many animals [1,2,3,4]. Transmission occurs by ingesting resistant cysts (*Blastocystis* sp., *B*. *coli*, and *G. duodenalis*) or oocysts (*Cryptosporidium* spp.) [5,6,7,8]. Infection can be direct (human-to-human, animal-to-human) or indirect when exposed to contaminated water and food [8]. Common symptoms and clinical signs of enteric protozoa found in humans and animals are acute diarrhea, abdominal pain, malabsorption, dehydration, anorexia, and weight loss [5,7,9,10]. However, asymptomatic infections can also occur frequently, depending on the health status of the host and also, particularly, on the protozoan species in question [3,11,12,13].

*Blastocystis* sp. is characterized by a lack of flagella [4]. This protozoan has a wide range of hosts, including humans, non-human primates, farm animals, and other wild mammals, such as artiodactyls, proboscideans, rodents, marsupials, birds, reptiles, amphibians, and insects [14]. The number of *Blastocystis* sp. infections is estimated to be 1 billion humans worldwide [15]. Its prevalence can vary from one country to another, with higher rates generally observed in developing countries [16], most likely due to poor hygiene habits and socioeconomic conditions [11]. Currently, there are at least 37 described subtypes (ST) of *Blastocystis* sp. The most common subtypes in humans are ST1–ST9, although a recent study has suggested the presence of ST12 in humans as well [14,17].

*Balantioides coli* is the only ciliate that infects humans [3]. Pigs are considered the main reservoirs, but other species, such as camels, cattle, donkeys, sheep, and goats, have been proposed as potential reservoirs [3,18]. Worldwide prevalence in humans typically does not exceed 1% [8]. The parasite has a direct life cycle, with a vegetative form, trophozoites, residing in the host intestine, and a resting cyst that is released into the environment in feces [18].

*Cryptosporidium* is a protozoan parasite of medical and veterinary importance that causes gastroenteritis in a variety of vertebrate species, including humans, as well as wild and domestic animals [2,9]. A recent study showed that *Cryptosporidium* infection was the fifth leading cause of diarrhea in children younger than five years, and acute infection caused more than 48,000 deaths [19]. Frequently reported risk factors include overcrowding, household diarrhea, poor-quality drinking water, animal contact, and open defecation/lack of toilet [20]. At least 44 *Cryptosporidium* species are considered taxonomically valid [21]. Of these, 19 are found in humans. *Cryptosporidium hominis* and *C. parvum* are responsible for approximately 95% of infections in humans, followed by *C. meleagridis*, *C. felis*, *C. canis*, *and C. ubiquitum.* Other species, such as *C. cuniculus*, *C. viatorum*, *C. muris*, *C. andersoni*, *C. erinacei*, *C. tyzzeri*, *C. bovis*, *C. suis*, *C. scrofarum*, *C. occultus*, *C. xiaoi*, *C. fayeri*, *C. ditrichi,* and the *Cryptosporidium* horse genotype, have also been found in humans [22,23,24].

*Giardia duodenalis* (syn *Giardia lamblia* and *Giardia intestinalis*) is a major cause of parasite-induced diarrheal disease infecting humans and animals [25]. Its life cycle consists of two stages: a fecally–orally transmitted cyst and a disease-causing trophozoite [26]. *G. duodenalis* is categorized into eight distinct assemblages (genotypes A–H), distinguished by host specificity and genetic variations [8,25]. Assemblages A and B specifically infect humans, and certain sub-genotypes exhibit zoonotic potential [25]. Following ingestion by a new host, acidic conditions in the host’s stomach facilitate excystation. Each cyst produces two trophozoites, which migrate to the duodenum and proximal jejunum. At this location, they adhere to the mucosal wall through a ventral adhesive disk and replicate via binary fission [5].

Hedgehogs are frequent dwellers of suburban environments. This proximity to humans raises the risk of exposure to infected hedgehog feces, particularly for individuals engaged in hedgehog rescue and rehabilitation efforts, providing the opportunity for zoonotic infections by enteric pathogens [27]. Hedgehogs can be hosts of enteric zoonotic protozoa such as *Blastocystis* sp., *Cryptosporidium* spp., and *G*. *duodenalis* [27,28,29,30]. Despite their potential role as hosts for enteric parasites, little data about their occurrence and molecular diversity are available in the scientific literature, with no studies having been conducted in Portugal. As such, this study aims to determine the occurrence and genetic diversity of *Blastocystis* sp., *B. coli*, *Cryptosporidium* spp., and *G*. *duodenalis* in hedgehogs from different areas of mainland Portugal.

## 2. Materials and Methods

### 2.1. Sample Collection

A total of 110 individual stool samples from hedgehogs were collected from Centro de Recuperação de Fauna do Parque Biológico de Gaia (CRFPBG; n = 30) and from Centro de Recuperação e Interpretação do Ouriço (CRIDO; n = 80), centers dedicated to rescuing hedgehogs from various regions, for further release into their natural environment.

**Figure 1:**
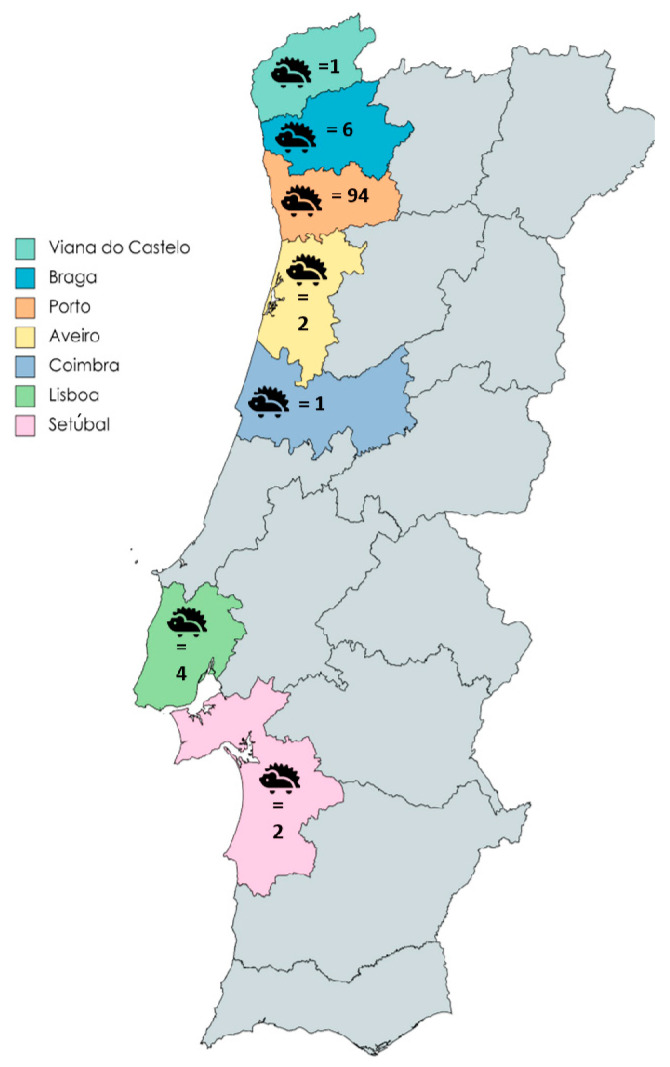
Geographic distribution of Portuguese hedgehogs tested for *Blastocystis* sp., *Balantioides coli*, *Cryptosporidium* spp., and *Giardia duodenalis*. Each district is distinguished by a unique color, followed by the corresponding number of hedgehogs obtained from from that district.

All hedgehog stool samples from the CRFPBG were from European hedgehogs (*Erinaceus europaeus*) and were obtained between September 2021 and April 2023. Stool samples from CRIDO included European hedgehogs, except for four individuals, namely three four-toed hedgehogs (*Atelerix albiventris*) and one long-eared hedgehog (*Hemiechinus auritus*). CRIDO samples were collected between December 2022 and May 2023. Most samples (n = 94) were derived from hedgehogs found in the Porto district, with smaller numbers from Braga (n = 6), Lisboa (n = 4), Setúbal (n = 2), Aveiro (n = 2), Coimbra (n = 1), and Viana do Castelo (n = 1) (Figure 1).

Stool samples were obtained from the cages where hedgehogs were kept, and all presented a well-structured texture indicative of absence of gastrointestinal disease. Importantly, no hedgehogs were sacrificed for the purpose of this study. The stool samples were quickly stored at 4 °C, maintained at this temperature during transportation to the laboratory, and then preserved at −20 °C until the DNA extraction process.

### 2.2. DNA Extraction

Fecal samples for DNA extraction were prepared by suspending fecal matter (10%) in phosphate-buffered saline (PBS) at pH 7.2. The suspensions were then centrifuged at 8000× *g* for 5 min. The supernatants were then used for DNA extraction and purification. DNA extraction and purification were performed using the QIAamp Cador Pathogen Mini Kit (Qiagen, Hilden, Germany) according to the manufacturer’s instructions. The QIAcube*^®^* automated platform (Qiagen) was used to process the extracted DNA. The eluted DNA was stored at −80 °C in RNase-free water.

### 2.3. Molecular Detection of Blastocystis sp., Balantioides coli, Cryptosporidium spp., and Giardia duodenalis

Specific molecular techniques were employed for the individual detection of *Blastocystis* sp., *Balantioides coli*, *Cryptosporidium* spp., and *Giardia duodenalis*. For *Blastocystis* sp., a direct PCR approach was employed, targeting a 600 bp segment of the SSU rRNA gene. This method followed the protocol described by Scicluna et al. (2006) [31] and utilized the primer set RD5/BhRDr with an annealing temperature of 59 °C.

For *Balantioides coli*, a direct PCR was performed targeting the complete ITS1–5.8s-rRNA–ITS2 region and the last 117 bp (3′ end) of the SSU rRNA gene, amplifying a 400 bp product. This procedure follows the method outlined by Ponce-Gordo et al. (2011) [32] and employs the primer set B5D/RD5 with an annealing temperature of 60 °C.

For identification of *Cryptosporidium* spp., a nested PCR was performed, targeting the 587 bp fragment of the SSU rRNA gene. This method utilized primer sets CR-P1/CRP2 and CR-P3/CPB-DIAGR for both rounds of amplification, maintaining a consistent annealing temperature of 50 °C, as described in Tiangtip and Jong-Wutiwes (2002) [33].

For *Giardia duodenalis*, a nested PCR approach was implemented. The initial RH11-derivates/Gia2150c primer pair was used to amplify a 497 bp product with an annealing temperature of 55 °C. Subsequently, a secondary RH11-derivates/RH4-derivates primer pair was employed to amplify a 293 bp fragment with an annealing temperature of 59 °C, replicating the strategy described in Helmy et al. (2018) [34].

### 2.4. PCR

The molecular detection of targeted parasites was performed through a series of PCR reactions, including endpoint, nested, and semi-nested protocols. Each PCR was carried out on the T100 thermocycler provided by Bio-Rad (Neuried, Germany). The reaction mixtures included the Fast PCR Mastermix (GRiSP*^®^*, Dortmund, Germany) and the 2× Xpert Fast Hotstart Mastermix (GRiSP*^®^*), ensuring optimal conditions for DNA amplification.

Following PCR amplification, DNA fragments, specific for each targeted parasite, were carefully separated and visualized through electrophoresis on 1.5% agarose gels. These gels were stained with Xpert Green Safe DNA gel dye (GRiSP*^®^*).

Electrophoresis was carried out at a constant voltage of 120 V for 30 min. For the confirmation of the results, the agarose gels were irradiated with UV light.

### 2.5. Statistical Analysis

Data processing and initial analysis were carried out using Microsoft^®^ Excel^®^ for Microsoft 365 MSO (version 2312 Build 16. 0. 17126. 20132) 64-bit. Descriptive analyses were conducted using IBM SPSS version 28.0.0.0 software for Windows (SPSS, Chicago, IL, USA). Throughout all analyses, a confidence interval (CI) of 95% was applied.

### 2.6. Sanger Sequencing and Phylogenetic Analysis

To isolate and purify amplicons, GRS PCR and Gel Band Purification Kit (GRiSP*^®^*) technology was employed. This method effectively separates positive amplicons that match the expected size from the surrounding non-target DNA fragments. Subsequently, the Sanger sequencing method was used, employing specific internal primers designed for the target gene. The bidirectional sequencing process generated two complementary sequences, which were then aligned and compared with existing entries in the NCBI (GenBank) nucleotide database. This comparative analysis was performed using the BioEdit Sequence Alignment Editor v7.1.9 software package (version 2.1), relying on the database accessed on 20 November 2023. For a more in-depth analysis and interpretation of the obtained sequences, the MEGA version X software was utilized [35]. Applying the Hasegawa–Kishino–Yano model, maximum likelihood (ML) bootstrap values with 1000 replicates were estimated for statistical robustness. The selection of this model, recognized as the most effective replacement, was determined through MEGA version X [35]. The sequences from this study have been deposited in the GenBank*^®^* database, with each assigned a unique accession number: OR835985 (*Blastocystis* sp.) and OR880424 (*Cryptosporidium parvum*).

## 3. Results

### Occurrence of Enteric Protozoan Parasites in Hedgehogs

From the analysis of the 110 stool samples for enteric protozoa, 1.82% (2/110 95% CI: 0.22–6.41) were positive for at least one protozoan. Among these, one sample (0.91% 1/110, 95% CI: 0.02–4.96) tested positive for *Blastocystis* and another for *Cryptosporidium* (0.91% 1/110, 95% CI: 0.02–4.96), as we can see in Table 1. All protozoa-positive stools were found exclusively in European hedgehogs from Centro de Recuperação de Fauna do Parque Biológico de Gaia in the Porto district of the northern region of mainland Portugal.

**Figure 2:**
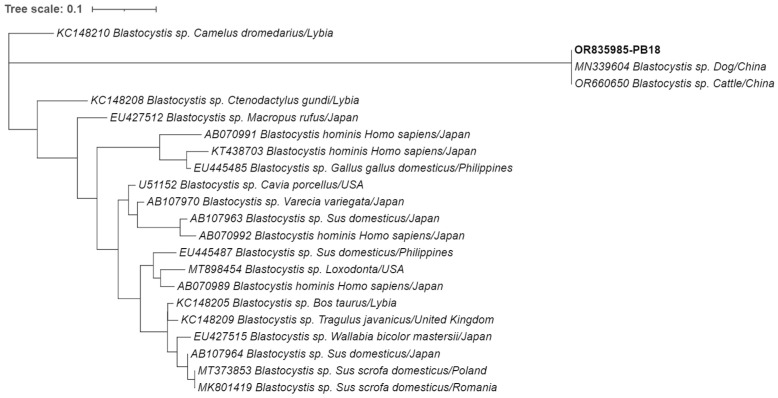
Phylogenetic analysis of the small-subunit ribosomal RNA (SSU-rRNA) gene was conducted for *Blastocystis* sp. found in European hedgehogs. Inference was performed using the maximum likelihood method and the Tamura 3-parameter model. The analyses were performed with the assistance of MEGA X software, followed by tree editing using Interactive Tree of Life (iTOL). The isolates obtained in this study are highlighted in bold with their respective accession numbers, while the 21 additional *Blastocystis* species strains obtained from GenBank are shown without bold formatting, followed by their respective accession numbers, species, hosts, and country of origin.

Regarding the characterization of *Blastocystis*, the phylogenetic tree illustrated in Figure 2 showed clustering of the sequence of *Blastocystis* sp. obtained in our study (highlighted in bold) with the sequences OR660650 and MN339604 isolated from a bovine and a dog in China, respectively. BLAST tool analysis established a 100% identity between these sequences. The reference sequence OR660650, originating from a bovine host, belongs to subtype ST3. This association, along with the 100% match, suggests that the sequence present in this study might also belong to the ST3 subtype.

**Figure 3:**
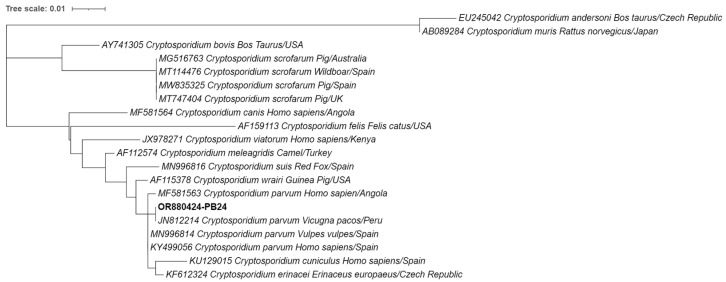
Phylogenetic analysis of the small-subunit ribosomal RNA (SSU-rRNA) gene was conducted for *Cryptosporidium* spp. found in European hedgehogs. Inference was performed using the maximum likelihood method and the Tamura 3-parameter model. The analyses were performed with the assistance of MEGA X software, followed by tree editing using Interactive Tree of Life (iTOL). The isolates obtained in this study are highlighted in bold with their respective accession numbers, while the 19 additional *Cryptosporidium* species strains obtained from GenBank are shown without bold formatting, followed by their respective accession numbers, species, hosts, and country of origin.

As for the genetic characterization of the detected *Cryptosporidium*, the phylogenetic analysis in Figure 3 and BLAST search revealed that the sequence obtained in this study exhibited 100% identity with a previously identified *C. parvum* sequence from an alpaca isolate in Peru (JN812214), suggesting that the sequence obtained in the current study represents a *C. parvum* strain.

## 4. Discussion

This study represents the first investigation performed in Portugal focusing on the molecular detection and phylogenetic analysis of intestinal protozoa in hedgehogs. Consequently, it represents the first report of *Blastocystis* and *Cryptosporidium* in hedgehogs from Portugal. *Blastocystis* sp. and *Cryptosporidium* spp. are among the most common agents responsible for enteric diseases globally, affecting both animals and humans [8]. Considering this information, the surveillance and identification of hedgehogs as possible hosts of these pathogenic protozoa is important for understanding the role of these agents.

In the present study, *Blastocystis* sp. subtype ST3 was identified in one hedgehog, representing an occurrence of 0.91% (1/110, 95% CI: 0.02–4.96). Noteworthily, *Blastocystis* sp. subtype ST3 has been previously found in humans [14]. There is a lack of information about the presence of *Blastocystis* in hedgehogs. To date and to the best of our knowledge, the only study available on the topic was conducted by Kaczmarek and Salamatin in 2018 [28] (Table 2), revealing an occurrence of 10.2%. It is crucial to note that the sample size in the previously mentioned study was approximately half of the size used in the current investigation. The identification of the subtype ST3 emphasizes the need for further research to understand the role of hedgehogs as possible reservoirs and their role in the transmission of *Blastocystis* sp. to both humans and animals.

*Cryptosporidium* was found in one isolate with the occurrence of 0.91% (1/110, 95% CI: 0.02–4.96). *Cryptosporidium* species have been reported in European hedgehogs such as *C. erinacei*, *C. parvum*, and *C. hominis* [27,36]. Compared to other studies performed with European hedgehogs (Table 3), the occurrence obtained in this study is lower than in those in the UK (8%) [37], Germany (29.8%) [30], Denmark (5.2%) [29], Czech Republic (73%) [36], and the Netherlands (9%) [27]. The disparities observed between our findings and previous research can be attributed to variations in sample size and the employment of diverse detection methods, including conventional microscopy and coproantigen detection techniques. Several studies have underscored the efficacy of molecular methods as highly sensitive and specific analytical tools, enabling simultaneous diagnosis and characterization of infections and offering more reliable data compared to serological and parasitological approaches [38]. Even when comparing studies employing PCR, the implementation of distinct oligonucleotide primers targeting different regions may contribute to disparities in sensitivities. The isolate was classified as *C. parvum*, one of the most common species of *Cryptosporidium* found in humans, with a global prevalence of 7.6% [39]. Given the synanthropic presence of hedgehogs in urban areas and their proximity to humans, concerns about their possible role as hosts for *C. parvum* should be considered. This underscores the need for further research, surveillance, and initiatives to mitigate the potential transmission of *C. parvum* from hedgehogs to humans and other animals.

Data on the occurrence of *G. duodenalis* and *B. coli* in European hedgehogs are limited. While the presence of *B. coli* has not yet been confirmed in hedgehogs, its recent detection in wild animals from Portugal, including wild boars [40] and wild cervids [41], suggests the potential for this parasite to exist in the country’s wildlife population. While *G. duodenalis* was not detected in the present study, its presence has been documented in other investigations. For instance, prevalence studies of *G. duodenalis* have been conducted in Germany, Russia, Denmark, New Zealand, and the Netherlands. The prevalence of *G. duodenalis* was found to be 0% in Germany, Russia, and Denmark, while the prevalence in New Zealand and the Netherlands was 33% and 11%, respectively (Table 4).

The detection of zoonotic enteric protozoan parasites, including *Blastocystis* and *Cryptosporidium,* could raise concerns for human and animal health. This emphasizes the need for monitoring and research to actively contain and prevent potential outbreaks in the hedgehog population.

## 5. Conclusions

In conclusion, this study represents the first investigation into the molecular detection and phylogenetic analysis of enteric protozoan parasites in European hedgehogs (*Erinaceus europaeus*) across mainland Portugal. This research showed a relatively low overall prevalence of these parasites within the hedgehog population. This study marks the initial identification of *Blastocystis* and *Cryptosporidium* in Portuguese hedgehogs, providing valuable data to the limited regional knowledge of these parasites. The detection of zoonotic parasites, particularly *Cryptosporidium* and *Blastocystis*, raises concerns about a possible transmission to humans. The surveillance, coupled with further research involving larger sample sizes and broader geographical representation, could be effective for the development of prevention and control strategies to mitigate the risk of outbreaks in hedgehog populations.

## Figures and Tables

**Table 1:** An overview of the occurrence of each enteric protozoan parasite in European hedgehogs (*Erinaceus europaeus*) from Portugal.

	Total (n)	Organism	Pos. (n)	Percentage (%)
European hedgehog (*Erinaceus europaeus*)	110	*Blastocystis* sp. (ST3)	1	0.91
		*Balantioides coli*	0	0
		*Cryptosporidium parvum*	1	0.91
		*Giardia duodenalis*	0	0

**Table 2:** Study on *Blastocystis* sp. in European hedgehogs (*Erinaceus europaeus*).

Country	Sample Size	Pos. (n)	Percentage (%)	Origin	Method	Subtype (n)	Reference
Poland	49	5	10.2	Wild	PCR	ST1 (4) ST7 (1)	[28]

PCR: polymerase chain reaction.

**Table 3:** Studies on *Cryptosporidium* spp. in European hedgehogs (*Erinaceus europaeus*).

Country	Sample Size	Pos. (n)	Percentage (%)	Origin	Methods	*Cryptosporidium* spp.	References
Czech Republic	15	11	73.3	Wild	CM/PCR	co-infection *C. parvum* + *C. erinacei*	[36]
Netherlands	90	8	9	Wild	PCR	*C. parvum* + *C. hominis*	[27]
UK	111	9	8	Wild	PCR	*C. parvum*	[37]
Germany	188	56	29.8	Wild	CADT	*C. parvum*	[30]
Denmark	268	14	5.2	Wild	CADT	*Cryptosporidium* spp.	[29]

CM: conventional microscopy; CADTs: copro-antigen detection techniques (ELISA, IFA, …); PCR: polymerase chain reaction.

**Table 4:** Studies on *Giardia duodenalis* in European hedgehogs (*Erinaceus europaeus*).

Country	Sample Size	Pos. (n)	Percentage (%)	Origin	Methods	Detected Genotypes	References
Germany	1175	0	0	Pet	CM	-	[42]
New Zealand	6	2	33	Wild	CM	-	[43]
Germany	106	0	0	Pet	CM	-	[44]
Germany	2	0	0	Pet	CADT	-	[45]
Netherlands	90	10	11	Wild	PCR	Assemblage A	[27]
Germany	205	0	0	Pet	CM	-	[46]
Russia	21	0	0	Pet	CM	-	[47]
Denmark	74	0	0	Wild	CADT	-	[29]

CM: conventional microscopy; CADTs: copro-antigen detection techniques (ELISA, IFA,…); PCR: polymerase chain reaction.

## Data Availability

The data presented in this study are available on request from the corresponding author.
